# Polymorphism of genes associated with infectious lung diseases in Northern Asian populations and in patients with community-acquired pneumonia

**DOI:** 10.18699/VJ21.51-o

**Published:** 2021-05

**Authors:** S.V. Mikhailova, L.V. Shcherbakova, N.I. Logvinenko, I.I. Logvinenko, M.I. Voevoda

**Affiliations:** Institute of Cytology and Genetics of the Siberian Branch of the Russian Academy of Sciences, Novosibirsk, Russia; Institute of Internal and Preventive Medicine – Branch of the Institute of Cytology and Genetics of the Siberian Branch of the Russian Academy of Sciences, Novosibirsk, Russia; Novosibirsk State Medical University, Novosibirsk, Russia; Institute of Internal and Preventive Medicine – Branch of the Institute of Cytology and Genetics of the Siberian Branch of the Russian Academy of Sciences, Novosibirsk, Russia Novosibirsk State Medical University, Novosibirsk, Russia; Institute of Cytology and Genetics of the Siberian Branch of the Russian Academy of Sciences, Novosibirsk, Russia

**Keywords:** community-acquired pneumonia, pulmonary tuberculosis, genetic predisposition, genetic polymorphism, TLR2, TIRAP, PKP3-SIGGIR-TMEM16J, long-lived people, внебольничная пневмония, туберкулез легких, генетическая предрасположенность, генетический полиморфизм, ген TLR2, ген TIRAP, генетический район PKP3-SIGGIR-TMEM16J, долгожители

## Abstract

The innate immune system is the first to respond to invading pathogens. It is responsible for invader recognition, immune-cell recruitment, adaptive-immunity activation, and regulation of inflammation intensity. Previously, two single-nucleotide polymorphisms of innate-immunity genes – rs5743708 (Arg753Gln) of the TLR2 gene and rs8177374 (Ser180Leu) of the TIRAP gene – have been shown to be associated with both pneumonia and tuberculosis in humans, but the data are contradictory among different ethnic groups. It has also been reported that rs10902158 at the PKP3-SIGGIR-TMEM16J genetic locus belongs to a haplotype race-specifically associated with tuberculosis. Meanwhile, a gradient of its frequency is observed in Asia. The aim of this work was to assess the effect of selection for the genotypes of the above-mentioned SNPs on the gene pools of populations living in harsh climatic conditions that contribute to the development of infectious lung diseases. We estimated the prevalence of these variants in white and Asian (Chukchis and Yakuts) population samples from Northern Asia and among patients with community-acquired pneumonia (CAP). Carriage of the rs5743708 A allele was found to predispose to severe CAP (odds ratio 2.77, p = 0.021), whereas the GG/CT genotype of rs5743708/rs8177374 proved to be protective against it (odds ratio 0.478, p = 0.022) in white patients. No association of rs10902158 with CAP (total or severe) was found among whites. Stratification of CAP by causative pathogen may help eliminate the current discrepancies between different studies. No significant difference in rs5743708 or rs8177374 was found between adolescent and long-lived white samples. Carriage of the alleles studied is probably not associated with predisposition to longevity among whites in Siberia. Both white and Asian populations studied were different from Western European and East Asian populations in the variants’ prevalence. The frequency of the rs8177374 T (Ser180Leu) variant was significantly higher in the Chukchi sample (p = 0, χ2 = 63.22) relative to the East Asian populations. This result may confirm the hypothesis about the selection of this allele in the course of human migration into areas with unfavorable climatic conditions.

## Abstract (ru)

: Врожденный иммунитет первым отвечает на инфекцию. Он участвует в распознавании патогена, привлечении к месту заражения иммунных клеток, а также активирует адаптивный иммунитет и регулирует интенсивность воспалительного ответа. Для двух однонуклеотидных полиморфизмов (ОНП) генов врожденного иммунитета – rs5743708 (Arg753Gln) гена TLR2 и rs8177374 (Ser180Leu) гена TIRAP – была показана ассоциация одновременно с пневмонией и туберкулезом, однако полученные данные различаются для разных этнических групп. Для rs10902158, расположенного в генетическом локусе PKP3-SIGGIR-TMEM16J, ранее было показано, что он входит в гаплотип, расоспецифически ассоциированный с туберкулезом. При этом на территории Азии наблюдается градиент его частоты. Целью нашей работы была оценка влияния отбора по генотипам названных ОНП на генофонды популяций, живущих в климатических условиях, неблагоприятных по инфекционным заболеваниям легких. Мы оценили распространение этих ОНП в выборках европеоидного и монголоидного (чукчи и якуты) населения Северной Азии и среди пациентов с внебольничной пневмонией. Носительство аллеля А rs5743708 гена TLR2 предрасполагало к развитию тяжелой внебольничной пневмонии (OR = 2.77, p = 0.021), а генотип GG/CT по rs5743708/rs8177374 оказался протективным против нее (OR = 0.478, p = 0.022) у европеоидов. Ассоциации rs10902158 с внебольничной пневмонией (как в целом, так и ее тяжелой формы) в европеоидной выборке обнаружено не было. Дифференцировка внебольничных пневмоний по их этиологии, возможно, позволит устранить наблюдаемые противоречия в данных разных исследователей об ассоциации исследованных ОНП с заболеванием. Не выявлено достоверных различий по частоте rs5743708 гена TLR2 и rs8177374 гена TIRAP между европеоидными выборками подростков и долгожителей. Вероятно, эти ОНП не влияют на предрасположенность к долгожительству в европеоидных популяциях, проживающих на Севере Евразии. Исследованные нами европеоидные и монголоидные популяционные выборки отличались по частотам вышеперечисленных вариантов от западноевропейских и восточноазиатских популяций. Частота варианта rs8177374 T (Ser180Leu) гена TIRAP в выборке чукчей была достоверно выше (p = 0, χ2 = 63.22), чем в популяциях Восточной Азии, что может служить подтверждением нашего предположения об отборе этого варианта в ходе миграции человека в районы с неблагоприятными климатическими условиями.

## Introduction

Innate immunity constitutes the first barrier against microorganisms and viruses by destroying infected cells and activating adaptive immunity. Nonetheless, an excessive nonspecific immune reaction (inflammation) may be life threatening because it can completely disrupt the functioning of vital organs. Community-acquired pneumonia (CAP) and pulmonary tuberculosis(PTB) are infectious diseases characterized by high mortality, and according to WHO, are ranked consistently among the top 10 leading causes of death in the world (https:// www.who.int/en/news-room/fact-sheets/detail/the-top-10causes-of-death).

Pneumonia is an inflammatory lower-respiratory-tract disease caused by viruses, bacteria, fungi, and parasites. In addition, it may be due to noninfectious processes or have a combined cause. For a long time, Streptococcus pneumoniae infection has been considered the main cause of CAP; however, it was shown recently that CAP develops mainly as a result of viral infections (influenza A and B viruses, parainfluenza viruses, adenovirus, respiratory syncytial virus, or coronaviruses) (Choi et al., 2012; Hong et al., 2014; Self et al., 2017). Streptococcus pneumoniae, Haemophilus inf luenzae, Staphylococcus aureus, Mycoplasma pneumoniae, Chlamydophila pneumoniae, Legionella pneumophila and other microbes may be causative agents of bacterial pneumonia (Choi et al., 2012; Hong et al., 2014; Self et al., 2017). After invasion of the airway epithelium by pathogens, these cells start to produce reactive oxygen species, cytokines, and other mediators to recruit immune cells. Being most abundant in lungs, alveolar macrophages ingest bacteria and apoptotic cells and can present antigens on MHC II to other immune cells. Proinflammatory M1 macrophages produce cytokines TNFα, IL-6, IL-1β, IL-12, and IL-23 to enhance inflammation for elimination of the invaders. Anti-inflammatory M2 macrophages produce cytokines IL-4, IL-13, and TGF-β to induce completion of the inflammatory reaction and remodeling of damaged tissue (Moldoveanu et al., 2009; Arango Duque, Descoteaux, 2014; Kumar, 2019).

Depending on the set of present chemokines and cytokines, different cells responsible for humoral and cellular immunity are attracted to the site of infection (Kumar, 2019). Severe pneumonias are more likely to develop in coinfections; it has been demonstrated that a viral infection (in particular influenza) facilitates the development of pneumococcal infection by damaging the epithelium and reducing the amount of a surfactant (McCullers, 2014; Aguilera, Lenz, 2020). Human respiratory syncytial virus, metapneumovirus, adenovirus, and influenza viruses A and B prefer a cold season (Price et al., 2019). The seasonal increase in the incidence of pneumococcal pneumonia coincides with seasonal outbreaks of influenza; S. pneumoniae, H. inf luenzae, and S. aureus infections have been reported to be associated with significant influenza pandemics (McCullers, 2014; Bystritskaya, Bilichenko, 2017; Morris et al., 2017). 

PTB is a pulmonary infectious disease caused mainly by Mycobacterium tuberculosis (Mtb). According to the WHO, approximately one-quarter of the world’s population is estimated to be infected by Mtb, and 5–15 % of these people will fall ill with active tuberculosis. In Russia, most of these patients (95 %) have PTB (https://minzdrav.gov.ru/ministry/ 61/22/stranitsa-979/statisticheskie-i-informatsionnye-mate rialy/statisticheskiy-sbornik-2018-god). The pathogenesis of pulmonary tuberculosis is based on Mtb survival after phagocytosis by alveolar macrophages. These bacteria can modulate a host immune response to protect the infected cells, change their metabolism, induce IL-10, suppress IL-12 and TNFα synthesis, and to inhibit MHC II expression and antigen presentation. Mtb makes macrophages unresponsive to interferon (IFN) γ and inhibits autophagy. It allows the mycobacteria to establish a persistent or latent infection in macrophages. Mycobacteria are believed to use the general mechanism of negative feedback regulation that restricts excessive inflammation (Harding, Boom, 2010; Richardson et al., 2015; Gopalakrishnan, Salgame, 2016). With the loss of immunity-driven control over mycobacterial reproduction, foamy macrophages accumulate in granulomas, and lung tissue necrosis begins (Liu C.H. et al., 2017). Vitamin D deficiency is known to negatively affect the effectiveness of the immune response in tuberculosis (Wilkinson et al., 2000; Aibana et al., 2019).

These data suggest that in Northern Asia, a region with low temperature and reduced insolation during most of the year, signs of purifying selection for genes associated with lung infections may be noticeable. For many genes of innate immunity, an association with viral, bacterial, and autoimmune diseases has been proven. Despite differences in the pathogenesis between CAP and PTB, it has been shown that minor alleles of rs5743708 (the TLR2 gene) and of rs8177374 (the TIRAP gene) can have a pathogenic and protective effect, respectively, in both of these lung diseases in humans. Nevertheless, data obtained by different research groups are contradictory.

Toll-like receptors (TLRs) play a pivotal role in host defense. Being membrane-anchored (TLRs 1, 2, 4–6, and 10) or endosomal (TLRs 3 and 7−9) in human immune cells (e.g., macrophages, monocytes, dendritic cells, and some leukocytes), they are involved in the recognition of structurally conserved surface molecules of microorganisms and viruses as well as viral nucleic acids (Barbalat et al., 2009; Kawai, Akira, 2010; Kumar, 2019). TLR2 participates in the recognition of a large number of diverse lipoproteins and peptidoglycans of gram-positive and gram-negative bacteria, fungi, and virus-infected cells. After ligand binding to the receptor, TIR (Toll-interleukin 1 receptor) domains of TLR2 and TLR1 or TLR2 and TLR6 dimerize via the formation of an extensive hydrogen-bonding network and hydrophobic interactions (Jin et al., 2007; Takeda, Akira, 2015). Homodimerization of the cytoplasmic domains of TLR2 does not induce TNFα production in vitro in murine macrophages, and the formation of the TLR2–TLR2 dimer is not detectable even in the presence of an agonist (Ozinsky et al., 2000; Shukla et al., 2018). Therefore, the existence of TLR2–TLR2 homodimers in vivo is being questioned. After ligand binding, reorientation of the TIR domains and triggering of a cascade of intracellular reactions lead to the activation of proinflammatory NF-κB and MAPK pathways, synthesis and a release of proinflammatory cytokines (IL-1β, IL-12, TNF-α, and IL-6) and various chemokines into extracellular space, and the development of an inflammatory response at the pathogen entry site (Liu C.H. et al., 2017; Tapader et al., 2018). In inflammatory monocytes, TLR2 induces type I IFN production in response to a viral ligand (Barbalat et al., 2009). It is reported that prolonged stimulation of TLR2 (more than 24 h) causes PI3K/Akt pathway activation in alveolar macrophages. It limits the production of NF-κB, TNF-α, and IL-12 and activates the synthesis of anti-inflammatory IL-10. This mechanism is assumed to prevent excessive inflammation (Richardson et al., 2015; Liu Y. et al., 2016).

The TLR2 gene is located in 4q31.3, has five exons, and expresses few splicing isoforms, but all of the coding sequences are contained within exon 3. The protein consists of 784 amino acid residues (aa) and includes extracellular leucine-rich repeat domains, which are primarily responsible for ligand recognition (aa 54–524), followed by the leucinerich repeat C-terminal domain (aa 525–579) and intracellular TIRdomain (aa 639–782), which mediates downstream signaling (https://www.uniprot.org/uniprot/O60603). It is expressed constitutively on macrophages and dendritic cells and can be induced in epithelial cells or B-cells. Its overexpression in patients with pneumococcal disease had been documented (Siebert et al., 2018)

Single-nucleotide polymorphism (SNP) rs5743708 (of the TLR2 gene) causing the Arg753Gln substitution is located in the TIR domain of the protein. This SNP is associated simultaneously with resistance to Lyme disease (Schröder et al., 2005) and with predisposition to tuberculosis (Guo, Xia, 2015; Patarčić et al., 2015), whereas the association with predisposition to PTB is race-specific (Caws et al., 2008; Guo, Xia, 2015; Hu et al., 2019). It is believed that TLR2 signaling may be nonessential to control acute tuberculosis but important during chronic tuberculosis (Gopalakrishnan, Salgame, 2016). Ameta-analysis has shown the TLR2 rs5743708 minor allele to be associated with CAP, Legionnaires’ disease, and pneumococcal disease; however, the data obtained in different studies are contradictory (Moens et al., 2007; Patarčić et al., 2015; Smelaya et al., 2016).

The TIRAP (TIR domain-containing adaptor protein) gene also known as Mal (MyD88 adapter-like) encodes one of the five adapter proteins that are involved in signal transduction from activated TLRs to protein kinases at the plasma membrane (Bonham et al., 2014). It is located in 11q24.2, consists of six exons, and encodes a protein of 221 aa. The TIRAP protein includes an N-terminal PEST domain (aa 15–35) responsible for binding to special sites in the plasma membrane, followed by an AB-loop mediating MyD88 and TLR4 binding. A binding site for TRAF6 (TNF receptor-associated factor 6) is located within the region aa 188–193 (Bernard, O’Neill, 2013). TIRAP is expressed in many cell types (Narayanan, Park, 2015), and its isoforms resulting from alternative splicing have unknown functions. There are different opinions about whether TIRAP forms a complex with the TIR domain of TLR6 for signal transmission; however, it has been proven that TIRAP mediates TLR2 and TLR4 signaling by facilitating the recruitment of the MyD88 adaptor protein to the TLRs (Nagpal et al., 2009; Bernard, O’Neill, 2013).

Activation of NF-κB, MAPK1, MAPK3, and JNK results in cytokine secretion and an inflammatory response. SNP rs8177374 (the TIRAP gene) is located in exon 5 and represents the Ser180Leu substitution in the encoded protein. It is located close to the TLR-binding site of TIRAP. In carriers of this substitution, modulation of TLR1, TLR2, TLR4, and TLR6 but not TLR9 signaling has been shown (Khor et al., 2007; Ferwerda et al., 2009; Siebert et al., 2018). Ser180Leu in a heterozygous state has a protective effect against PTB and invasive pneumococcal disease in white and African samples and against malaria in African and Asian samples (Khor et al., 2007; Panda et al., 2016). Carriage of heterozygous Ser180Leu protects children from pneumococcal lower-respiratory-tract infections, whereas carriers of the homozygous 180Leu polymorphism alone or in combination with some TLR1 and TLR6 polymorphisms may be susceptible to recurrent pneumococcal infections (Siebert et al., 2018). Simultaneous carriage of the TIRAP 180Leu variant and some SNPs in the TLR4 gene as well as 180Leu homozygosity increases susceptibility to severe hospital-acquired infections (Kumpf et al., 2010).

The opposite results have been obtained as well. The rs8177374 T allele (180Leu) increases the risk of PTB in a sample of Iranian population (Naderi et al., 2014). A metaanalysis of nine published case-control studies did not reveal a significant association of 180L with tuberculosis risk (Miao et al., 2011). There are controversial opinions about the mechanism behind the observed protective effect of Ser180Leu heterozygosity. They are based on differences in observed effects of the SNP at the level of proinflammatory cytokines. Depending on the model used, some research groups showed an increased level (Ferwerda et al., 2009; Panda et al., 2016) and others a decreased level (Khor et al., 2007; Kumpf et al., 2010; Siebert et al., 2018) of cytokines after their induction in 180 Leu/Leu carriers. Accordingly, homozygosity of the minor variant of rs8177374 is thought to cause either an excessive inflammatory reaction or the absence of an adequate immune response. It is supposed that selection pressure on the TIRAP gene provides a balance between protection against excessive inflammation and effective defense during infectious diseases (Khor et al., 2007; Ferwerda et al., 2009).

Besides the polymorphisms in genes TLR2 and TIRAP, in this paper, we focused on the PKP3-SIGGIR-TMEM16J gene region. An association of its haplotypes with different types of tuberculosis has been shown among children in Vietnam and South Africa (Horne et al., 2012; Gupta et al., 2016). It is believed that the impact of the haplotypes on immunity is determined by SIGIRR (single immunoglobulin interleukin 1 receptor; synonym: IL-1R8), which is a negative regulator of TLR signaling (Molgora et al., 2016). Carriage of rs10902158 GG and rs7111432 AA in introns of PKP3 and TMEM16J, respectively, acts additively with a vitamin D deficiency and “pathogenic” genotypes of rs5743708 (TLR2) and rs8177374 (TIRAP) on tuberculosis predisposition (Horne et al., 2012; Gupta et al., 2016). rs10902158 located in intron 2 of the PKP3 gene has been analyzed. Encoded desmosomal plaque protein plakophilin 3 is involved in intracellular adhesion (Gurjar et al., 2018). Of note, rs10902158 has a frequency gradient in Asia; according to the Genome Aggregation Database (GnomAD) (https://gnomad.broadinstitute. org/), it is absent in South Asia and is found with a frequency of ~50 % in Southeast Asia. Nonetheless, functional significance of genetic variants in noncoding parts of the PKP3-SIGGIRTMEM16J gene region, including rs10902158, is not clear.

In this work, we analyzed the frequencies of rs5743708, rs8177374, and rs10902158 (for which conflicting data on the association with respiratory infections have been reported previously) in white and Asian samples from Northern Asia and among CAP patients. According to the statistics of the Ministry of Health of the Russian Federation, Novosibirsk Oblast and Yakutia are characterized by an increased incidence of PTB, whereas Chukotka Autonomous Okrug is the leader in both pneumonia and PTB morbidity in Russia (https://minzdrav.gov.ru/ministry/61/22/stranitsa-979/ statisticheskie-i-informatsionnye-materialy/statisticheskiysbornik-2017-god; https://minzdrav.gov.ru/ministry/61/22/ stranitsa-979/statisticheskie-i-informatsionnye-materialy/ statisticheskiy-sbornik-2018-god). According to the WHO, the highest death rate from pneumonia is observed before the age of 5 and after 75–80 years (https://www.who.int/medicines/ areas/priority_medicines/Ch6_22Pneumo.pdf). Therefore, we assumed that long-lived people of the Siberian Federal District may differ from adolescents in the frequency of rs5743708 and rs8177374, and we assessed the prevalence of the pathogenic variants in the sample of long-lived people.

## Materials and methods

The study protocol was approved by the local Ethics Committee of the Institute of Internal and Preventive Medicine (branch of the Institute of Cytology and Genetics of the Siberian Branch of the Russian Academy of Sciences, Novosibirsk, Russia; approval No. 22.06.2008). Written informed consent to be examined and to participate in the study was obtained from each patient. For individuals younger than 18 years, the informed consent was signed by a parent or legal guardian.

The white sample consisted of 451 adolescents (95 % Russians, 197 males, 253 females, aged 14–17) from Oktiabr’skii district of Novosibirsk (55°01′ N 82°55′ E) and 289 Russian settlers (120 males, 169 females, aged 45–64, mean age 52.5) in towns Tommot (58°58′00″ N 126°16′0″ E), Neryungri (56°39′30″N 124°43′30″ E), Ust-Nera (64°34′05″ N 143°14′10″ E), and Yakutsk (62°01′38″ N 129°43′55″ E), who lived in Yakutia for more than 16 years or were born there. The adolescent sample was described previously (Zavyalova et al., 2011). Asian samples (220 individuals) consisted of 130Chukchis (66 males, 64 females, aged 18–73, mean age 40) from the Kanchalan village (65°10′41″ N 176°44′52″ E) of Chukotka Autonomous Okrug and 132 ­Yakuts (53 males, 79 females, aged 44–64, mean age 49) from the Kylayy village (63°13′34″ N 132°08′06″ E) and towns Tommot, Neryungri, and Ust-Nera of Yakutia. The sample of long-lived people was collected in cities Novosibirsk, Tomsk, and Tumen and consisted of 188 individuals (180 females, 8 males) aged 90– 105, mean age 92.

Ethnicity of individuals was identified using questionnaires and additional cross-examination with elucidation of the nationality of ancestors (at least in three generations). Persons of mixed origin were excluded from the analysis. The CAP patient sample (406 whites) was collected in offices of pulmonary hospitals of Novosibirsk and Yakutsk in 2003–2005 before the COVID-19 outbreak. The sample consists of 120 patients with severe CAP (aged 18–80, mean age 52) and 286 patients with nonsevere (mild to moderate severity) CAP (aged 16–92, mean age 39). The diagnosis of pneumonia was made on the basis of radiologically confirmed “fresh” lung tissue infiltration and clinical data (fever, cough, sputum production, chest pain, and shortness of breath) in the absence of an obvious diagnostic alternative. CAP of various etiologies was regarded as severe if the CURB65 rating scale index was 4–5 points (Lim et al., 2003).

Genomic DNA was extracted from peripheral blood leucocytes by the standard phenol–chloroform method (Sambrook, Russell, 2006). Genotyping was performed using polymerase chain reaction (PCR) with an analysis of restriction fragment length polymorphism by electrophoresis in a 5 % polyacrylamide gel after visualization with an ethidium bromide solution.

The rs5743708 SNP (TLR2) was identified by amplification of DNA with primers 5′-GCCATTCTCATTCTTCTGG* AGC-3′ and 5′-GGGAACCTAGGACTTTATCGCA-3′ (* denotes a nucleotide changed for restriction site creation). The 168-bp PCR product was digested with the Pst I restriction endonuclease (SibEnzyme, Novosibirsk) for 2 h at 37 °C. The rs5743708 A (753Q) allele was revealed by the presence of fragments of 20, 45, and 103 bp, whereas the rs5743708 G allele by fragments of 20 and 148 bp.

The detection of rs8177374 (TIRAP) was performed by amplification of genomic DNA with primers 5′-GGCTGC ACCATCCCCCA*GC-3′ and 5′-CCGTTCCCCTTCTCCCT 

CCTGTAG-3′ (* denotes a nucleotide changed for restriction site creation). The 162-bp PCR product was digested with the AccB7 I restriction endonuclease (SibEnzyme, Novosibirsk) for 2 h at 37 °C. The rs8177374 T (180L) allele was identified by the presence of fragments of 21 and 141 bp, whereas in case of rs8177374 C, the PCR product was not cut.

Primers 5′-TGGCAAGGATTGGAGAACTC*C*TGTC-3′ and 5′-CAGGGCCAGTGCCTCCCC-3′ (* denotes nucleotides changed for restriction site creation) were used for the amplification of the PKP3 intron 2 sequence containing rs10902158. The resulting 192-bp amplicon was digested with the BstEN I restriction endonuclease (Sibenzyme, Novosibirsk) for 2 h at 65 °C. In the presence of the rs10902158 А allele, the PCR product was not cut, whereas in the case of the rs10902158 G allele, fragments of 24 and 168 bp were observed.

Statistical analysis was performed in the SPSS 16.0 software.

## Results

Genotype distributions were consistent with the Hardy–Weinberg equilibrium among all the population samples (data not shown). Minor allele frequencies for rs5743708, rs8177374, and rs10902158 are represented in Table 1.

**Table 1.**
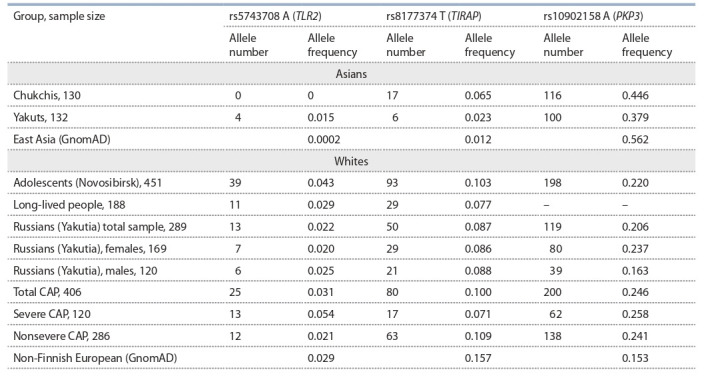
Minor allele frequencies for rs5743708, rs8177374, and rs10902158 in the studied samples and in the Genome Aggregation Database (GnomAD) Note: CAP, community-acquired pneumonia

In the sample of Novosibirsk adolescents, allele frequencies of rs5743708, rs8177374, and rs10902158 differed from those of the non-Finnish European sample in GnomAD (χ2 = 6.621, p = 0.013; χ2 = 19.541, p = 0; χ2 = 54.554, p = 0, respectively).

p = 0.013; χ2 = 19.541, p = 0; χ2 = 54.554, p = 0, respectively). For the frequency of the rs5743708 A (Arg753Gln) allele, there was a tendency for a decrease in Russian settlers in Yakutia and among long-lived people compared with the Novosibirsk sample.

The rs8177374 T (Ser180Leu) frequency did not differ among the studied white samples

In the sample of Russian settlers of Yakutia, males and females differed in the frequency of rs10902158 ( p = 0.037, χ2 = 4.86). Moreover, the frequency of this SNP among males was closer to that observed in non-Finnish Europeans according to GnomAD data, and the frequency among females was closer to that observed in the Novosibirsk sample. Perhaps there were sex differences during recent migration to Yakutia from different regions of Russia. Genetic analysis of a larger sample and estimation of this SNP’s frequency in western regions of Russia are required for explaining the observed differences

The two analyzed Asian samples differed from each other and from GnomAD East Asian cohorts. Among Yakuts, the rs5743708A (Arg753Gln) variant, which is very rare in other Asian populations, was found at a frequency of 0.015±0.007 (mean±SD). Chukchis differed significantly from GnomAD East Asians in rs10902158 allele frequency (p = 0, χ2 = 63.22) (see Table 1).

Frequencies of polymorphisms rs5743708, rs8177374, and rs10902158 were not different among adolescents and total white CAP patient samples. By contrast, after the sample was divided into patients with severe and nonsevere CAP, differences were found for rs5743708 (p = 0.021, χ2 = 6.24). Next, genotype frequencies were estimated for rs5743708, rs8177374, and rs10902158 in the examined samples (except for long-lived people regarding rs10902158). For the latter SNP (in the PKP3 gene), no difference in frequency was detectable within any group (data not shown). The observed distribution of rs5743708 and rs8177374 genotypes among the studied samples is presented in Table 2.

**Table 2.**
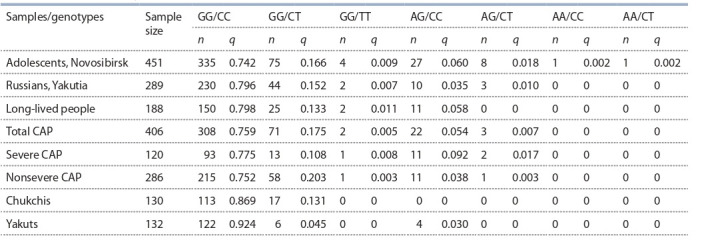
Combined genotype frequencies of rs5743708 and rs8177374 in the studied populations and patient groups Note: n, number of genotype carriers; q, genotype frequency.

The frequencies of genotypes of rs5743708 and rs8177374 did not differ among the following samples: adolescents of Novosibirsk, long-lived people of Siberia, all patients with CAP, and all Russians in Yakutia. Possibly, the carriage of the studied alleles is not associated with predisposition to longevity in Siberia and does not significantly affect the probability of resettling of migrants in more unfavorable climatic conditions at present. This notion is consistent with WHO findings that in Eastern Europe, in contrast to Western and Central Europe, the pneumonia mortality rate does not increase significantly after age 80 (https://www.who.int/medicines/areas/ priority_medicines/Ch6_22Pneumo.pdf). As for pneumonia, carriage of the rs5743708 A allele predisposed to severe CAP (AG/CC+AG/CT vs all: odds ratio 2.77, 95 % confidence interval 1.227–6.272, p = 0.021). The heterozygous genotype of rs8177374 in combination with the GG genotype of rs5743708 had a protective effect against severe CAP (GG/CT vs all: odds ratio 0.478, 95 % confidence interval 0.251–0.909, p = 0.022).

## Discussion

It was shown here that carriage of none of the three studied SNPs, rs5743708, rs8177374, and rs10902158, is associated with the predisposition to CAP in total. By contrast, we found that the Arg753Gln variant of TLR2 predisposes to severe CAP, and the heterozygous Ser180Leu variant of TIRAP in combination with the 753 Arg/Arg variant of TLR2 has a protective effect against it in the white population. These data partially explain the contradictions in the data from different researchers. Most likely, the contribution of the alleles of genes TLR2 and TIRAP to CAP predisposition is determined by pneumonia etiology. A substantial proportion of severe pneumonia cases are known to be caused by combined viral and bacterial infections (McCullers, 2014; Morris et al., 2017; Aguilera, Lenz, 2020). TLR2 is responsible mainly for the recognition of bacteria-associated molecular patterns; Ser180Leu of the TIRAP gene modulates signal transduction only from TLR2 and TLR4 recognizing molecular patterns of bacteria as well (Nagpal et al., 2009). Most likely, combined and bacterial but not viral pneumonias are associated with TLR2 and TIRAP gene variants.

The studied Asian ethno-geographical groups showed an increased frequency of the protective rs8177374 T (Ser180Leu) variant of TIRAP relative to neighboring East Asian populations. In the Chukchi sample, the difference was significant (p = 0, χ2 = 63.22). It may be a consequence of the natural selection that has promoted protection from excessive inflammation during pulmonary diseases. The hypothesis about the selection of the Ser180Leu variant along with the out-ofAfrica migration to a harsh environment has been advanced earlier (Khor et al., 2007; Ferwerda et al., 2009). An increased frequency of the Arg753Gln variant of TLR2 and a decreased frequency of Ser180Leu of TIRAP as compared to non-Finnish Europeans may indicate higher genetic predisposition of the Siberian white population to PTB and severe CAP. Nevertheless, there are a lot of genes associated with CAP and PTB independently. Apparently, during the settlement of peoples in Northern Eurasia, the formation of gene pools had been determined by the selection that facilitated adaptation to specific infections (Lime disease among them), parasites, and the climate. It would be interesting to determine why two mutations changing the same TLR2 signaling have opposite effects on the predisposition to severe CAP. One possible explanation is the difference in the structure and functions of heterodimers TLR2–TLR1 and TLR2–TLR6 (see the Figure).

**Fig. 1.**
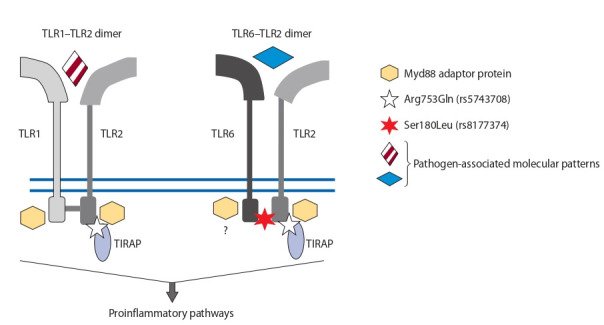
Locations of the Arg753Gln substitution in TLR2 and Ser180Leu in TIRAP on the protein complexes.

Existing data on the roles of TLRs 1, 2, and 6 in the activation of proinflammatory and anti-inflammatory signaling are conflicting. Overexpression of TLR2 carrying the Arg753Gln variant has been demonstrated to cause a significantly stronger impairment of cytokine induction by TLR2/TLR1 ligands as compared with TLR2/TLR6 ligands in the HEK293 cell line (Schröder et al., 2005). In later papers, it has been shown that the Arg-to-Gln substitution at position 753 of TLR2 changes the size, charge, and hydrophobic properties of this site and reduces the ability of TLR2 to form a heterodimer with TLR6 (Basith et al., 2011; Xiong et al., 2012). This SNP significantly alters agonist-inducible association of TLR2 with adaptor proteins TIRAP and MyD88 and impairs NF-κB signaling and IL-8 mRNA expression in the HEK293 cell line (Xiong et al., 2012). Genes TLR1 and TLR2 have different expression activators (Lancioni et al., 2011). In the TLR2–TLR1 dimer, TLR1 and TLR2 are responsible for NF-κB and MAPK pathways and for PI3K pathway activation, respectively; therefore, upregulation of proinflammatory cytokines is TLR1-dependent, whereas upregulation of type I IFN is TLR2-dependent (Raieli et al., 2019). TLR2–TLR6 binding disruption possibly causes an increase in the number of TLR2–TLR1 dimers in which TLR1 drives the activation of the NF-κB inflammatory signaling cascade. On the contrary, the Ser180Leu variant of TIRAP weakens proinflammatory signal transmission. Besides, TIRAP acts as an adaptor protein for TLR4 homodimers. This receptor is primarily responsible for the recognition of lipopolysaccharides of gram-negative bacteria and fungi (Takeda, Akira, 2015). It is believed that TLR4 takes part not only in MyD88-dependent proinf lammatory signaling but also in MyD88-independent anti-inf lammatory signaling. Weakening of TLR4 signaling through TIRAP probably enhances the signaling through adapter proteins TRIF and TRAM, causing the secretion of anti-inf lammatory cytokines (Li et al., 2013).

It remains unclear why the SNPs in TLR2 and TIRAP have similar effects on the predisposition to or protection against acute (CAP) and chronic (PTB) lung infections. Perhaps this phenomenon is due to an impact on inflammation in both diseases. The severity of CAP is determined by life-threatening acute inflammation; in PTB, the development of chronic inflammation masks the infection from the host immune system (Liu C.H. et al., 2017).

## Conclusion

In Northern Asian populations, the observed difference in rs8177374 frequency may reflect consequences of natural selection during the settlement of peoples on territories with unfavorable climatic conditions. As for pneumonia, carriage of the rs5743708 A allele (the TLR2 gene) predisposes to severe CAP; the heterozygous genotype of rs8177374 (the TIRAP gene) in combination with the GG genotype of rs5743708 (the TLR2 gene) has a protective effect against it. Stratification of CAP by causative pathogen may help to eliminate the current discrepancies among research groups. Regional differences in a set of pathogens along with the genetic characteristics of ethno-geographical groups can determine the associations of various genetic variants of innate immunity with the prevalence and severity of pneumonia.

## Conflict of interest

The authors declare no conflict of interest.

## References

Aguilera E.R., Lenz L.L. Inflammation as a modulator of host susceptibility to pulmonary influenza, pneumococcal, and co-infections.
Front. Immunol. 2020;11:105. DOI 10.3389/fimmu.2020.00105.

Aibana O., Huang C.C., Aboud S., Arnedo-Pena A., Becerra M.C., Bellido-Blasco J.B., Bhosale R., Calderon R., Chiang S., Contreras C.,
Davaasambuu G., Fawzi W.W., Franke M.F., Galea J.T., Garcia-Ferrer D., Gil-Fortuño M., Gomila-Sard B., Gupta A., Gupte N., Hussain R., Iborra-Millet J., Iqbal N.T., Juan-Cerdán J.V., Kinikar A.,
Lecca L., Mave V., Meseguer-Ferrer N., Montepiedra G., Mugusi F.M., Owolabi O.A., Parsonnet J., Roach-Poblete F., Romeu-García M.A., Spector S.A., Sudfeld C.R., Tenforde M.W., Togun T.O.,
Yataco R., Zhang Z., Murray M.B. Vitamin D status and risk of incident tuberculosis disease: a nested case-control study, systematic
review, and individual-participant data meta-analysis. PLoS Med.
2019;16(9):e1002907. DOI 10.1371/journal.pmed.1002907.

Arango Duque G., Descoteaux A. Macrophage cytokines: involvement
in immunity and infectious diseases. Front. Immunol. 2014;5:491.
DOI 10.3389/fimmu.2014.00491.

Barbalat R., Lau L., Locksley R.M., Barton G.M. Toll-like receptor 2
on inflammatory monocytes induces type I interferon in response
to viral but not bacterial ligands. Nat. Immunol. 2009;10(11):12001207. DOI 10.1038/ni.1792.

Basith S., Manavalan B., Govindaraj R.G., Choi S. In silico approach
to inhibition of signaling pathways of Toll-like receptors 2 and 4 by
ST2L. PLoS One. 2011;6(8):e23989. DOI 10.1371/journal.pone.
0023989.

Bernard N.J., O’Neill L.A. Mal, more than a bridge to MyD88. IUBMB
Life. 2013;65(9):777-786. DOI 10.1002/iub.1201.

Bonham K.S., Orzalli M.H., Hayashi K., Wolf A.I., Glanemann C.,
Weninger W., Iwasaki A., Knipe D.M., Kagan J.C. A promiscuous lipid-binding protein diversifies the subcellular sites of tolllike receptor signal transduction. Cell. 2014;156(4):705-716. DOI
10.1016/j.cell.2014.01.019.

Bystritskaya E.V., Bilichenko T.N. An analysis of pneumonia morbidity in adults and children at Russian Federation, 2010–2014.
Pulmonologiya = Russian Pulmonology. 2017;27(2):173-178. DOI
10.18093/0869-0189-2017-27-2-173-178. (in Russian)

Caws M., Thwaites G., Dunstan S., Hawn T.R., Lan N.T., Thuong N.T.,
Stepniewska K., Huyen M.N., Bang N.D., Loc T.H., Gagneux S.,
van Soolingen D., Kremer K., van der Sande M., Small P., Anh P.T.,
Chinh N.T., Quy H.T., Duyen N.T., Tho D.Q., Hieu N.T., Torok E.,
Hien T.T., Dung N.H., Nhu N.T., Duy P.M., van Vinh Chau N., FarrarJ. The influence of host and bacterial genotype on the development
of disseminated disease with Mycobacterium tuberculosis. PLoS
Pathog. 2008;4(3):e1000034. DOI 10.1371/journal.ppat.1000034.

Choi S.H., Hong S.B., Ko G.B., LeeY., Park H.J., Park S.Y., Moon S.M.,
Cho O.H., Park K.H., Chong Y.P., Kim S.H., Huh J.W., Sung H.,
Do K.H., Lee S.O., Kim M.N., Jeong J.Y., Lim C.M., Kim Y.S.,
Woo J.H., Koh Y. Viral infection in patients with severe pneumonia
requiring intensive care unit admission. Am. J. Respir. Crit. Care
Med. 2012;186(4):325-332. DOI 10.1164/rccm.201112-2240OC.

Ferwerda B., Alonso S., Banahan K., McCall M.B.B., GiamarellosBourboulis E.J., Ramakers B.P., Mouktaroudi M., Fain P.R., Izagirre N., Syafruddin D., Cristea T., Mockenhaupt F.P., Troye-Blomberg M., Kumpf O., Maiga B., Dolo A., Doumbo O., Sundaresan S.,
Bedu-Addo G., van CrevelR., Hamann L., Oh D.-Y., SchumannR.R.,
Joosten L.A.B., de la Rúa C., Sauerwein R., Drenth J.P.H., Kullberg B.-J., van der Ven A.J.A.M., Hill A.V., Pickkers P., van der
Meer J.W.M., O’Neill L.A.J., Netea M.G. Functional and genetic
evidence that the Mal/TIRAP allele variant 180L has been selected
by providing protection against septic shock. Proc. Natl. Acad. Sci.
USA. 2009;106(25):10272-10277. DOI 10.1073/pnas.0811273106.

Gopalakrishnan A., Salgame P. Toll-like receptor 2 in host defense
against Mycobacterium tuberculosis: to be or not to be – that is the
question. Curr. Opin. Immunol. 2016;42:76-82. DOI 10.1016/j.coi.
2016.06.003.

Guo X.G., Xia Y. The rs5743708 gene polymorphism in the TLR2
gene contributes to the risk of tuberculosis disease. Int. J. Clin. Exp.
Pathol. 2015;8(9):11921-11928.

Gupta A., Montepiedra G., Gupte A., Zeldow B., Jubulis J., Detrick B.,
Violari A., Madhi S., Bobat R., Cotton M., Mitchell С., Spector S.,
IMPAACT NWCS113 and P1041 Study Team. Low vitamin-D
levels combined with PKP3-SIGIRR-TMEM16J host variants is associated with tuberculosis and death in HIV-infected and -exposed
infants. PLoS One. 2016;11(2):e0148649. DOI 10.1371/journal.
pone.0148649.

Gurjar M., Raychaudhuri K., Mahadik S., Reddy D., Atak A., Shetty T.,
Rao K., Karkhanis M.S., Gosavi P., Sehgal L., Gupta S., Dalal S.N.
Plakophilin3 increases desmosome assembly, size and stability by
increasing expression of desmocollin2. Biochem. Biophys. Res.
Commun. 2018;495(1):768-774. DOI 10.1016/j.bbrc.2017.11.085.

Harding C.V., Boom W.H. Regulation of antigen presentation by Mycobacterium tuberculosis: a role for Toll-like receptors. Nat. Rev.
Microbiol. 2010;8(4):296-307. DOI 10.1038/nrmicro2321.

Hong H.L., Hong S.B., Ko G.B., Huh J.W., Sung H., Do K.H.,
Kim S.H., Lee S.O., Kim M.N., Jeong J.Y., Lim C.M., Kim Y.S.,
Woo J.H., Koh Y., Choi S.H. Viral infection is not uncommon in
adult patients with severe hospital-acquired pneumonia. PLoS One.
2014;9(4):e95865. DOI 10.1371/journal.pone.0095865.

Horne D.J., Randhawa A.K., Chau T.T., Bang N.D., Yen N.T., Farrar J.J., Dunstan S.J., Hawn T.R. Common polymorphisms in the
PKP3-SIGIRR-TMEM16J gene region are associated with susceptibility to tuberculosis. J. Infect Dis. 2012;205(4):586-594. DOI
10.1093/infdis/jir785.

Hu L., Tao H., Tao X., Tang X., Xu C. TLR2 Arg753Gln gene polymorphism associated with tuberculosis susceptibility: an updated
meta-analysis. Biomed. Res. Int. 2019;2628101. DOI 10.1155/2019/
2628101.

Jin M.S., Kim S.E., Heo J.Y., Lee M.E., Kim H.M., Paik S.G., Lee H.,
Lee J.O. Crystal structure of the TLR1–TLR2 heterodimer induced
by binding of a tri-acylated lipopeptide. Cell. 2007;130(6):10711082. DOI 10.1016/j.cell.2007.09.008.

Kawai T., Akira S. The role of pattern-recognition receptors in innate immunity: update on Toll-like receptors. Nat. Immunol. 2010;
11(5):373-384. DOI 10.1038/ni.1863.

Khor C.C., Chapman S.J., Vannberg F.O., Dunne A., Murphy C.,
Ling E.Y., Frodsham A.J., Walley A.J., Kyrieleis O., Khan A.,
Aucan C., Segal S., Moore C.E., Knox K., Campbell S.J., Lienhardt C., Scott A., Aaby P., Sow O.Y., Grignani R.T., Sillah J.,
Sirugo G., Peshu N., Williams T.N., Maitland K., Davies R.J.,
Kwiatkowski D.P., Day N.P., Yala D., Crook D.W., Marsh K.,
Berkley J.A., O’Neill L.A., Hill A.V. A Mal functional variant is
associated with protection against invasive pneumococcal disease,
bacteremia, malaria and tuberculosis. Nat. Genet. 2007;39(4):523528. DOI 10.1038/ng1976.

Kumar V. Inflammation research sails through the sea of immunology
to reach immunometabolism. Int. Immunopharmacol. 2019;73:128145. DOI 10.1016/j.intimp.2019.05.002.

Kumpf O., Giamarellos-Bourboulis E.J., Koch A., Hamann L., Mouktaroudi M., Oh D.Y., Latz E., Lorenz E., Schwartz D.A., Ferwerda B., Routsi C., Skalioti C., Kullberg B.J., van der Meer J.W.,
Schlag P.M., Netea M.G., Zacharowski K., Schumann R.R. Influence
of genetic variations in TLR4 and TIRAP/Mal on the course of sepsis
and pneumonia and cytokine release: an observational study in three
cohorts. Crit. Care. 2010;14(3):R103. DOI 10.1186/cc9047.

Lancioni C.L., Li Q., Thomas J.J., Ding X., Thiel B., Drage M.G., Pecora N.D., ZiadyA.G., Shank S., Harding C.V., Boom W.H., Rojas R.E.
Mycobacterium tuberculosis lipoproteins directly regulate human
memory CD4+ T cell activation via Toll-like receptors 1 and 2.
Infect. Immun. 2011;79(2):663-673. DOI 10.1128/IAI.00806-10.

Li J., Lee D.S., Madrenas J. Evolving bacterial envelopes and plasticity of TLR2-dependent responses: basic research and translational
opportunities. Front. Immunol. 2013;4:347. DOI 10.3389/fimmu.
2013.00347.

Lim W.S., van der Eerden M.M., Laing R., Boersma W.G., Karalus N.,
Town G.I., Lewis S.A., Macfarlane J.T. Defining community acquired pneumonia severity on presentation to hospital: an international derivation and validation study. Thorax. 2003;58:377-382.
DOI 10.1136/thorax.58.5.377.

Liu C.H., Liu H., Ge B. Innate immunity in tuberculosis: host defense
vs pathogen evasion. Cell Mol. Immunol. 2017;14(12):963-975.
DOI 10.1038/cmi.2017.88.

Liu Y., Li J.Y., Chen S.T., Huang H.R., Cai H. The rLrp of Mycobacterium tuberculosis inhibits proinflammatory cytokine production and
downregulates APC function in mouse macrophages via a TLR2mediated PI3K/Akt pathway activation-dependent mechanism. Cell.
Mol. Immunol. 2016;13(6):729-746. DOI 10.1038/cmi.2015.58.

McCullers J.A. The co-pathogenesis of influenza viruses with bacteria
in the lung. Nat. Rev. Microbiol. 2014;12(4):252-262. DOI 10.1038/
nrmicro3231.

Miao R., Li J., Sun Z., Xu F., Shen H. Meta-analysis on the association
of TIRAP S180L variant and tuberculosis susceptibility. Tuberculosis (Edinb.). 2011;91(3):268-272. DOI 10.1016/j.tube.2011.01.006.

Moens L., Verhaegen J., Pierik M., Vermeire S., De Boeck K., Peetermans W.E., Bossuyt X. Toll-like receptor 2 and Toll-like receptor 4
polymorphisms in invasive pneumococcal disease. Microbes Infect.
2007;9(1):15-20. DOI 10.1016/j.micinf.2006.10.002.

Moldoveanu B., Otmishi P., Jani P., Walker J., Sarmiento X., Guardiola J., Saad M., Yu J. Inflammatory mechanisms in the lung.
J. Inf lamm. Res. 2009;2:1-11.

Molgora M., Barajon I., Mantovani A., Garlanda C. Regulatory role of
IL-1R8 in immunity and disease. Front. Immunol. 2016;7:149. DOI
10.3389/fimmu.2016.00149.

Morris D.E., Cleary D.W., Clarke S.C. Secondary bacterial infections
associated with influenza pandemics. Front. Microbiol. 2017;8:1041.
DOI 10.3389/fmicb.2017.01041.

Naderi M., Hashemi M., Pourmontaseri Z., Eskandari-Nasab E., Bahari G., Taheri M. TIRAP rs8177374 gene polymorphism increased
the risk of pulmonary tuberculosis in Zahedan, southeast Iran.
Asian Pac. J. Trop. Med. 2014;7(6):451-455. DOI 10.1016/S19957645(14)60073-0.

Nagpal K., Plantinga T.S., Wong J., Monks B.G., Gay N.J., Netea M.G.,
Fitzgerald K.A., Golenbock D.T. A TIR domain variant of MyD88 adapter-like (Mal)/TIRAP results in loss of MyD88 binding and reduced TLR2/TLR4 signaling. J. Biol. Chem. 2009;284(38):2574225748. DOI 10.1074/jbc.M109.014886.

Narayanan K.B., Park H.H. Toll/interleukin-1 receptor (TIR) domainmediated cellular signaling pathways. Apoptosis. 2015;20(2):196209. DOI 10.1007/s10495-014-1073-1.

Ozinsky A., Underhill D.M., Fontenot J.D., Hajjar A.M., Smith K.D.,
Wilson C.B., Schroeder L., Aderem A. The repertoire forpattern recognition of pathogens by the innate immune system is defined by
cooperation between Toll-like receptors. Proc. Natl. Acad. Sci. USA.
2000;97:13766-13771. DOI 10.1073/pnas.250476497.

Panda A.K., Das B.K., Panda A., Tripathy R., Pattnaik S.S., Mahto H.,
Pied S., Pathak S., Sharma S., Ravindran B. Heterozygous mutants
of TIRAP (S180L) polymorphism protect adult patients with Plasmodium falciparum infection against severe disease and mortality.
Infect. Genet. Evol. 2016;43:146-150. DOI 10.1016/j.meegid.2016.
04.035.

Patarčić I., Gelemanović A., Kirin M., Kolčić I., Theodoratou E., Baillie K.J., de Jong M.D., Rudan I., Campbell H., Polašek O. The
role of host genetic factors in respiratory tract infectious diseases:
systematic review, meta-analyses and field synopsis. Sci. Rep. 2015;
5:16119. DOI 10.1038/srep16119.

Price R.H.M., Graham C., Ramalingam S. Association between viral
seasonality and meteorological factors. Sci. Rep. 2019;9(1):929.
DOI 10.1038/s41598-018-37481-y.

Raieli S., Trichot C., Korniotis S., Pattarini L., Soumelis V. TLR1/2 orchestrate human plasmacytoid predendritic cell response to gram +
bacteria. PLoS Biol. 2019;17(4):e3000209. DOI 10.1371/journal.
pbio.3000209.

Richardson E.T., Shukla S., Sweet D.R., Wearsch P.A., Tsichlis P.N.,
Boom W.H., Harding C.V. Toll-like receptor 2-dependent extracellular signal-regulated kinase signaling in Mycobacterium tuberculosis-infected macrophages drives anti-inflammatory responses and
inhibits Th1 polarization of responding T cells. Infect. Immun. 2015;
83(6):2242-2254. DOI 10.1128/IAI.00135-15.

Sambrook J., Russell D.W. Purification of nucleic acids by extraction
with phenol:chloroform. Cold Spring Harbor Protoc. 2006;2006(1):
4455. DOI 10.1101/pdb.prot4455.

Schröder N.W.J., Diterich I., Zinke A., Eckert J., Draing C., von
Baehr V., Hassler D., Priem S., Hahn K., Michelsen K.S., Hartung T., Burmester G.R., Göbel U.B., Hermann C., Schumann R.R.
Heterozygous Arg753Gln polymorphism of human TLR-2 impairs
immune activation by Borrelia burgdorferi and protects from late
stage Lyme disease. J. Immunol. 2005;175(4):2534-2540. DOI
10.4049/jimmunol.175.4.2534.

Self W.H., Balk R.A., Grijalva C.G., Williams D.J., Zhu Y., Anderson E.J., Waterer G.W., Courtney D.M., Bramley A.M., Trabue C.,
Fakhran S., Blaschke A.J., Jain S., Edwards K.M., Wunderink R.G.
Procalcitonin as a marker of etiology in adults hospitalized with
community-acquired pneumonia. Clin. Infect. Dis. 2017;65(2):183190. DOI 10.1093/cid/cix317.

Shukla S., Richardson E.T., Drage M.G., Boom W.H., Harding C.V.
Mycobacterium tuberculosis lipoprotein and lipoglycan binding
to Toll-like receptor 2 correlates with agonist activity and functional outcomes. Infect. Immun. 2018;86(10). pii: e00450-18. DOI
10.1128/IAI.00450-18.

Siebert J.N., Hamann L., Verolet C.M., Gameiro C., Grillet S.,
Siegrist C.A., Posfay-Barbe K.M. Toll-interleukin 1 receptor domain-containing adaptor protein 180L single-nucleotide polymorphism is associated with susceptibility to recurrent pneumococcal
lower respiratory tract infections in children. Front. Immunol. 2018;
9:1780. DOI 10.3389/fimmu.2018.01780.

Smelaya T.V., Belopolskaya O.B., Smirnova S.V., Kuzovlev A.N.,
Moroz V.V., Golubev A.M., Pabalan N.A., Salnikova L.E. Genetic
dissection of host immune response in pneumonia development and
progression. Sci. Rep. 2016;6:35021. DOI 10.1038/srep35021.

Takeda K., Akira S. Toll-like receptors. Curr. Protoc. Immunol. 2015;
109:14.12.1-14.12.10. DOI 10.1002/0471142735.im1412s109.

Tapader R., Bose D., Dutta P., Das S., Pal A. SslE (YghJ), a cell-associated and secreted lipoprotein of neonatal septicemic Escherichia coli, induces Toll-like receptor 2-dependent macrophage
activation and proinflammation through NF-kB and MAP kinase
signaling. Infect. Immun. 2018;86(9):e00399-18. DOI 10.1128/IAI.
00399-18.

Wilkinson R.J., Llewelyn M., Toossi Z., Patel P., Pasvol G., Lalvani A., Wright D., Latif M., Davidson R.N. Influence of vitamin D
deficiency and vitamin D receptor polymorphisms on tuberculosis among Gujarati Asians in west London: a case-control study.
Lancet. 2000;355(9204):618-621. DOI 10.1016/S0140-6736(99)
02301-6.

Xiong Y., Song C., Snyder G.A., Sundberg E.J., Medvedev A.E. R753Q
polymorphism inhibits Toll-like receptor (TLR) 2 tyrosine phosphorylation, dimerization with TLR6, and recruitment of myeloid
differentiation primary response protein 88. J. Biol. Chem. 2012;
287(45):38327-38337. DOI 10.1074/jbc.M112.375493.

Zavyalova L.G., Denisova D.V., Simonova G.I., Orlov P.S., Voevoda M.I. Association of polymorphisms of genes FTO and TCF7L2
with cadiometabolic parameters of the adolescents in Siberia. Bulleten SO RAMN = Bulleten SB RAMS. 2011;31(5):5-13. (in Russian).

